# Mental Health Interest and Its Prediction during the COVID-19 Pandemic Using Google Trends

**DOI:** 10.3390/ijerph182312369

**Published:** 2021-11-24

**Authors:** Magdalena Sycińska-Dziarnowska, Liliana Szyszka-Sommerfeld, Karolina Kłoda, Michele Simeone, Krzysztof Woźniak, Gianrico Spagnuolo

**Affiliations:** 1 Department of Orthodontics, Pomeranian Medical University in Szczecin, 70-111 Szczecin, Poland; magdadziarnowska@gmail.com (M.S.-D.); liliana.szyszka@gmail.com (L.S.-S.); krzysztof.wozniak@pum.edu.pl (K.W.); 2 MEDFIT Karolina Kłoda, 71-050 Szczecin, Poland; wikarla@gazeta.pl; 3 Department of Neurosciences, Reproductive and Odontostomatological Sciences, University of Naples “Federico II”, 80131 Napoli, Italy; michele.simeone@unina.it; 4 Institute of Dentistry, I. M. Sechenov First Moscow State Medical University, 119435 Moscow, Russia

**Keywords:** mental health, depression, insomnia, loneliness, psychologist, psychiatrist, COVID-19, Google Trends

## Abstract

This study aimed to analyze and predict interest in mental health-related queries created in Google Trends (GT) during the COVID-19 pandemic. The Google Trends tool collected data on the Google search engine interest and provided real-time surveillance. Five key phrases: “depression”, “insomnia”, ”loneliness”, “psychologist”, and “psychiatrist”, were studied for the period from 25 September 2016 to 19 September 2021. The predictions for the upcoming trend were carried out for the period from September 2021 to September 2023 and were estimated by a hybrid five-component model. The results show a decrease of interest in the search queries “depression” and “loneliness” by 15.3% and 7.2%, respectively. Compared to the period under review, an increase of 5.2% in “insomnia” expression and 8.4% in the “psychiatrist” phrase were predicted. The expression “psychologist” is expected to show an almost unchanged interest. The upcoming changes in the expressions connected with mental health might be explained by vaccination and the gradual removal of social distancing rules. Finally, the analysis of GT can provide a timely insight into the mental health interest of a population and give a forecast for a short period trend.

## 1. Introduction

The COVID-19 pandemic is described as a major public health burden worldwide [1]. As of October 2021, there have been more than 242 million confirmed COVID-19 cases and almost 5 million deaths due to COVID-19 associated disease. Despite more than 6 billion doses of vaccine administered, the virus continues to spread [2,3]. Since the outburst of the global COVID-19 pandemic, we have faced a unique situation that has affected mental health. Quarantine, isolation, and dramatic news reports contributed to a perceived increase in anxiety and decline in mood [4,5,6]. One of the first early scientific reviews on the psychological impact of quarantine published in Lancet highlighted the importance of clear and effective information directed at the population to limit the perceived stress and adverse mental effects resulting mainly from the restriction of liberty [5].

Quarantine and isolation, in the pandemic setting, are defined as the separation of people diagnosed with an infectious disease from those who are not sick. The uncertainty and social isolation during the pandemic could lead to mental and physical health disorders [7,8]. The separation and restriction of movement required from individuals during the COVID-19 pandemic have the potential to cause stress and depression. Sleep deprivation and insomnia have been a focus of scientific study as responses to stressful situations such as natural disasters or war [9]. The COVID-19 pandemic is now considered a phenomenon characterized not simply by a series of limitations but with high levels of psychological stress, which may be clinically significant and include anxiety, post-traumatic stress disorder, and depression [10].

Google Trends (GT) is gaining popularity for exploring online social behavior and predicting disease outbreaks and pandemics. This novel tool has attracted a larger scientific community, and Trends analyses have been used in various scientific fields to predict relevant medical, social, and political findings [11,12,13]. As the situation during the COVID-19 pandemic changes rapidly, a real-time data analysis would be helpful to assess the current information about public interest and concerns. Moreover, in the future, there will be a need to assess the mental health consequences of the pandemic and how they can be mitigated [14]. In the era of the COVID-19 pandemic, the ability of GT to track interest in mental health in the larger population group is important and was analyzed and described in [15,16]. However, given the scarcity of research into the global changes in mental status during the COVID-19 pandemic, this study aimed to detect and predict the impact of the COVID-19 disease on population mental health.

## 2. Materials and Methods

The data for this study was collected from the open GT database among anonymous Google search engine users [17,18]. In total, five data collections were imported according to the chosen time period—from 25 September 2016 to 19 September 2021. Each data observation represents a weekly number of queries processed. Every sample was collected by inscribing exclusively one special key phrase: “depression”, “insomnia”, ”loneliness”, “psychologist”, and “psychiatrist”. The sample names for the whole world reflect such phrases whenever it is possible. Every sample is normalized, ranging from 0 to 100, where 0 stands for lack of requests and 100 stands for maximum requests in a stated period.

All statistical estimations along with data visualization were programmed into the interpreted programming language “R” [19] with the open-source for data science, scientific research, and technical communication software “R studio” version 1.4.1106 (RStudio, PBC: Boston, MA, USA, 2020) [20]. The data analysis was carried out by using the R v.4.1.1 software environment (codenamed “Kick Things”) and RStudio IDE v. 1.4.1717 (codenamed “Juliet Rose”). During the analysis, several R packages were used, e.g., “ggplot2” [21] for data visualization and “dplyr” [22] for data manipulation. At the forecasting stage, several built-in functions from *forecast* [23,24,25] and *forecastHybridR* [26] packages were used. The predicted values were estimated by the hybrid five-component model. Hybrid models have the fundamental advantage of combining two or more individual models, which means that the models have the potential of complementing each other. For this reason, the five-component hybrid model is able to exploit the advantages of each individual model’s characteristics [27].

The following five models (components) were included in the ensemble:

- The best ARIMA model for univariate time series (auto arima) [25]. The ARIMA model is a generalization of the autoregressive moving average (ARMA) model. ARIMA forecasting is obtained by inserting time series data for the variable of interest. The stationarity check step is performed immediately after identifying the appropriate number of lags or amount of differencing to be applied to the data. The results are often interpreted similarly to the multiple linear regression model [28].
- The theta method model (thetam). The Theta method of forecasting, proposed by Assimakopoulos and Nikolopoulos [29], is a special case of exponential smoothing with drift. Due to its simplicity, it is in high demand among forecast practitioners and has high accuracy in forecasting time series of various characters and different frequencies [30]. Its performance was admitted in M3-Competition [31], where it performed much better than the participating advanced methods and expert systems, and it outperformed the rest of competitors, particularly for monthly series and microeconomic data.
- Feed-forward neural networks with a single hidden layer and lagged inputs for forecasting univariate time series (nnetar). Neural Network Auto-Regression Model (NNETAR) is a neural network, and parametric nonlinear model applied to forecasting tasks [25]. In the NNETAR, after determining the order of the auto-regressive model, the neural network is trained by the training dataset by considering the auto-regressive order. The number of input nodes or time series lags of the neural network is determined by the autoregressive order [32].
- Seasonal Loess’ decomposition of time series obtained by applying a nonseasonal forecasting method to the seasonally adjusted data and re-seasonalizing it using the last year of the seasonal component (stlm). The Stlm takes a time series y, applies the Seasonal and Trend decomposition using Loess (STL decomposition), and models the seasonally adjusted data using a model passed in as a model function or specified using method. It returns an object that includes the original STL decomposition and a time series model fitted to the seasonally adjusted data [33].
- Exponential smoothing State–Space model with Box–Cox transformation, ARMA errors, Trend, and Seasonal components (tbats) [34]. The Box–Cox Transformation is used to deal with nonlinear data, and the ARMA model for residuals can de-correlated the time series data. TBATS model can improve the prediction performance compared to the simple State–Space Model. The trigonometric expression of seasonality terms does not only dramatically reduce the model parameters when the seasonality frequencies are high but also gives the model more flexibility in dealing with complex seasonality [33].

In order to increase the prediction accuracy, the model component weights were selected between three adjusted models: by applying the hybrid model with the equal component’s weights (M1), with weighting by in-sample error (M2), with weighting by in-sample error with the hyperparameter tuning (M3). The following hyperparameters were used for the tuning procedure:

1.  max.p = 12, max.q = 12 without approximation for *auto arima* component;
2.  the 50 time repeats for *nnetar* component;
3.  a true robust option for *stlm* component;
4.  without using arma errors for *tbats* component.

The comparison of the above models and the choosing of the final one was made based on accuracy measures, among which the mean absolute percentage error (MAPE) was chosen as arbitrary.

In order to calculate prediction intervals with the required coverage, first, the covariance estimation between the different forecast errors was conducted, after which the resulting variance expression for the linear combination of component’s weights of the model with the lowest MAPE was performed.

## 3. Results

### 3.1. Depression

An accuracy measures for M1, M2, M3 models for “depression” data were estimated and presented in Table 1.

As the lowest MAPE measure was achieved by conducting the M2 model, it was used as a final model for further predictions.

**Figure 1:**
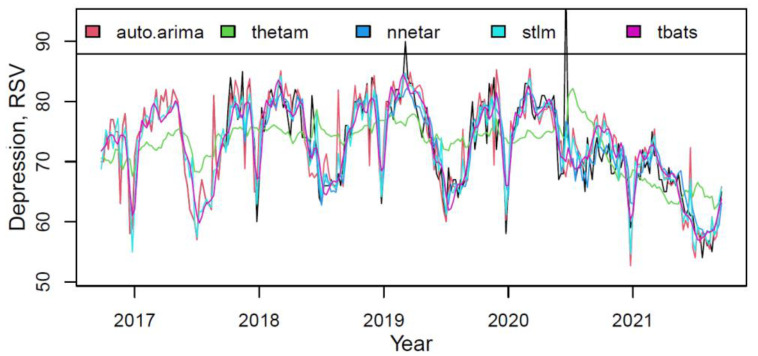
The actual (black) and fitted values of each component of the M2 hybrid model.

The plot in Figure 1 presented the actual and fitted values for each component of the M2 model on the “depression” training data.

Most models described data fairly accurately (only the *thetam* had the perceptible underfitting). The model component’s weights, based on the quality of fitting the training data, are shown in Table 2.

**Figure 2:**
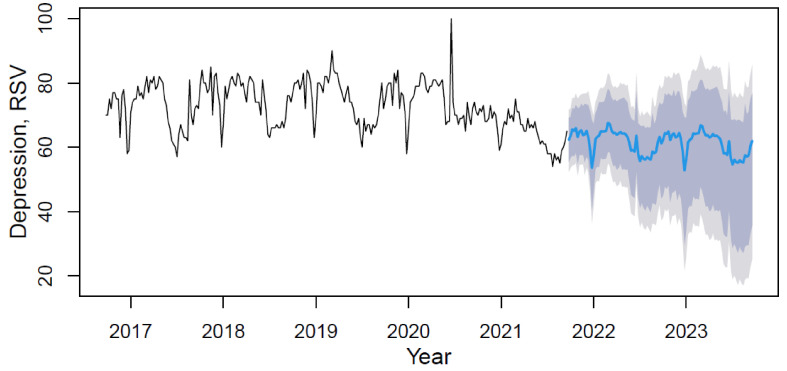
The depression’s data time series plot (solid black line) with forecast (solid blue line) on light grey (95% CI) and dark grey (80% CI) backgrounds by applying the M2 hybrid model.

Using the M2 model with weights from Table 2, data forecasting was conducted with its visualization in Figure 2.

Based on the forecast results, the future trend can be characterized as slightly negative with rhythmic seasonality. The mean value of the “depression” indicator in the forecast period (from September 2021 to September 2023) will decrease in comparison with the period under review by 15.3%.

### 3.2. Insomnia

An accuracy measures for M1, M2, M3 models for “insomnia” data were estimated and presented in Table 3.

As the lowest MAPE measure was achieved by conducting the M3 model, this model was used as the final one for further predictions.

**Figure 3:**
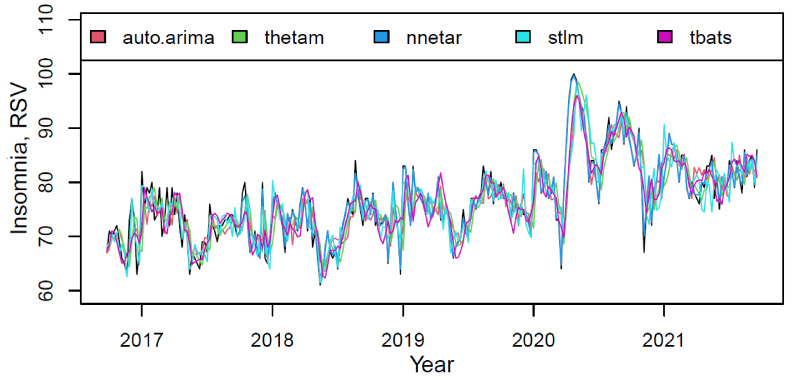
The actual (black) and fitted values of each component of the M3 hybrid model.

The plot in Figure 3 presented the actual and fitted values for each component of the M3 model on the “insomnia” training data.

The models described data fairly accurately. However, compared to *nnetar*, the rest have a slight underfitting. The model components weights, based on the quality of fitting the training data, are shown in Table 4.

**Figure 4:**
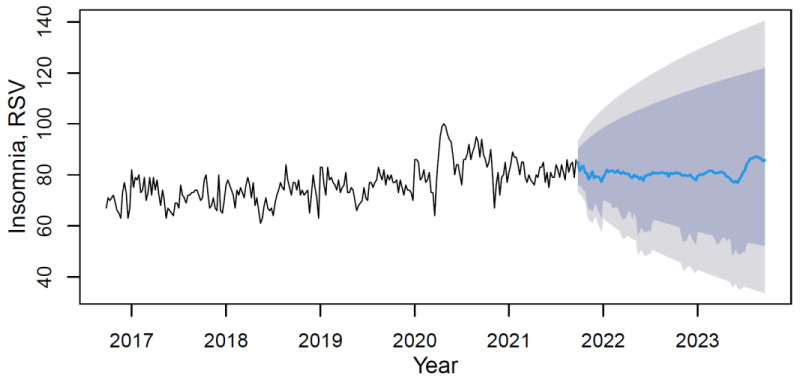
Insomnia’s data time series plot (solid black line) with forecast (solid blue line) on light grey (95% CI) and dark grey (80% CI) backgrounds by applying the M3 hybrid model.

Using the M3 model with weights from Table 4, data forecasting was conducted with its visualization in Figure 4.

Based on the forecast results, the future trend can be characterized as steady positive without visible seasonality. The mean value of the “insomnia” indicator in the forecast period (from September 2021 to September 2023) will increase compared to the period under review by 5.2%.

### 3.3. Loneliness

An accuracy measures for M1, M2, M3 models for loneliness data were estimated and presented in Table 5.

As the lowest MAPE measure was achieved by conducting the M3 model, this model was used as the final one for further predictions.

**Figure 5:**
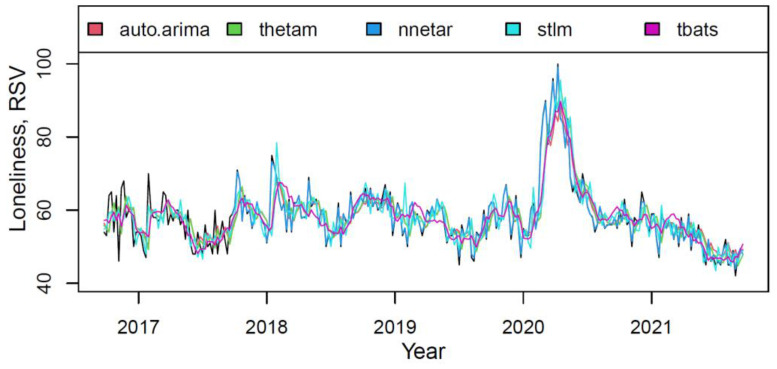
The actual (black) and fitted values of each component of the M3 hybrid model.

The plot in Figure 5 presented the actual and fitted values for each component of the M3 model on the “loneliness” training data.

The models described data fairly accurately (especially for the first year). However, compared to *nnetar*, the rest have a slight underfitting.

The model components weights, based on the quality of fitting the training data, are shown in Table 6.

**Figure 6:**
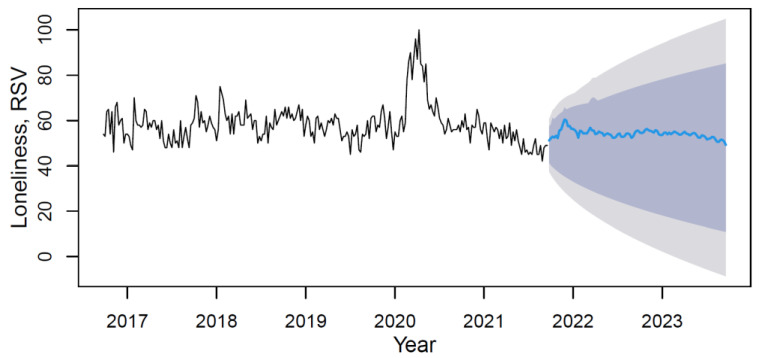
The loneliness’s data time series plot (solid black line) with forecast (solid blue line) on light grey (95% CI) and dark grey (80% CI) backgrounds by applying the M3 hybrid model.

Using the M3 model with weights from Table 6, data forecasting was conducted with its visualization in Figure 6.

Based on the forecast results, the future trend can be characterized as slightly negative without visible seasonality. The mean value of the “loneliness” indicator in the forecast period (from September 2021 to September 2023) will decrease in comparison with the period under review by 7.2%.

### 3.4. Psychologist

An accuracy measures for M1, M2, M3 models for “psychologist” data were estimated and presented in Table 7.

As the lowest MAPE measure was achieved by conducting the M3 model, this model was used as the final one for further predictions.

**Figure 7:**
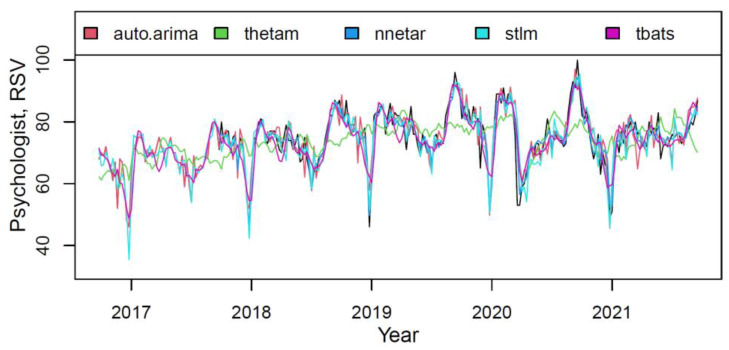
The actual (black) and fitted values of each component of the M3 hybrid model.

The plot in Figure 7 presented the actual and fitted values for each component of the M3 model on the “psychologist” training data.

Most models described data fairly accurately (only the thetam had the perceptible underfitting). The model components weights, based on the quality of fitting the training data, are shown in Table 8.

**Figure 8:**
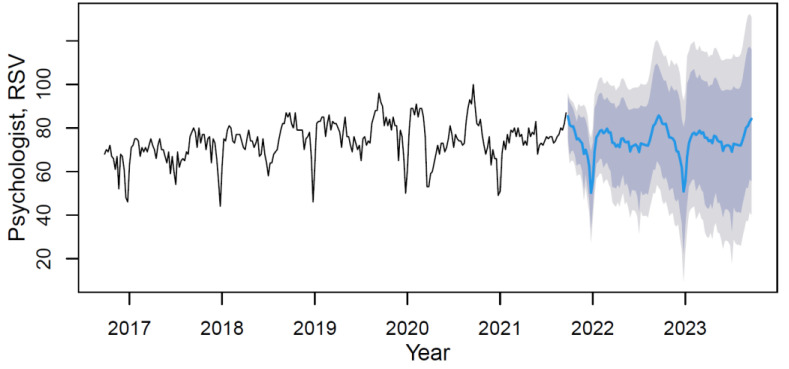
The psychologist’s data time series plot (solid black line) with forecast (solid blue line) on light grey (95% CI) and dark grey (80% CI) backgrounds) by applying the M3 hybrid model.

Using the M3 model with weights from Table 8, data forecasting was conducted with its visualization in Figure 8.

Based on the forecast results, the trend can be characterized as steady with rhythmic seasonality. The mean value of the “psychologist” indicator in the forecast period (from September 2021 to September 2023) will not change significantly (0.2%) and will remain within the boundaries of the statistical error.

### 3.5. Psychiatrist

An accuracy measures for M1, M2, M3 models for “psychiatrist” data were estimated and presented in Table 9.

As the lowest MAPE measure was achieved by conducting the M3 model, this model was used as the final one for further predictions.

**Figure 9:**
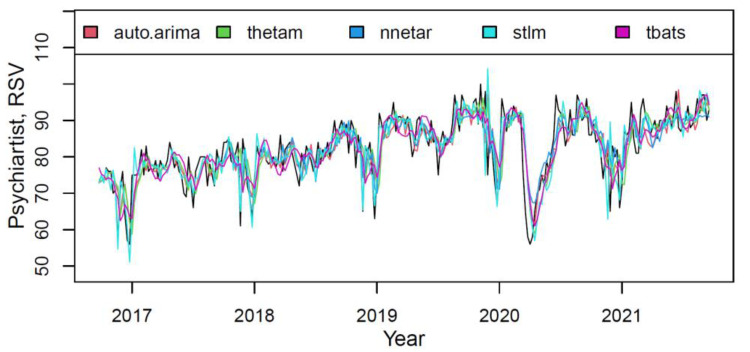
The actual (black) and fitted values of each component of the M3 hybrid model.

The plot in Figure 9 presented the actual and fitted values for each component of the M3 model on the “psychiatrist” training data.

Most models described data reasonably well (however, one can notice insignificant under fittings of *thetam*, *nnetar* and on the contrary, lesser *stlm*’s overfittings). The model component’s weights, based on the quality of fitting the training data, are shown in Table 10.

**Figure 10:**
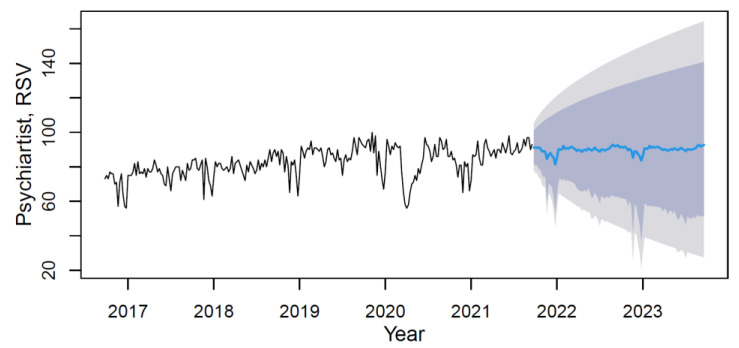
The psychiatrist’s data time series plot (solid black line) with forecast (solid blue line) on light grey (95% CI) and dark grey (80% CI) backgrounds by applying the M3 hybrid model.

By applying the M3 model with weights from Table 10, data forecasting was conducted with its visualization in Figure 10.

Based on the forecast results, the future trend can be characterized as steady with rhythmic low-magnitude seasonality. The mean value of the “psychiatrist” indicator in the forecast period (from September 2021 to September 2023) will increase in comparison with the period under review by 8.4%. Given the relatively stable values of the forecasted RSV in 2021–2023, the main reason for the growth lies in the significantly lower comparative indicators in the period until 2019.

## 4. Discussion

The purpose of this study was to investigate and predict the impact of the COVID-19 pandemic on population mental health. In our study, changes in search terms related to mental health during the COVID-19 pandemic were observed. Queries about “depression” were high during the first spring quarantine in 2020; however, in the carried out forecast, this expression is predicted to decrease between September 2021 and September 2023 by 15.3%. The decrease in “depression” queries was also noted in the study conducted by Reger et al. The authors explain it as the outcome of social coherence and the use of adaptive coping strategies [35]. In the study conducted from 27 April to 13 May 2020, as many as 50.9% of participants showed signs of anxiety, 57.4% of respondents suffered from stress, and 58.6% showed features of depression. According to the authors, stress and depression are globally prevalent during the COVID-19 pandemic [36]. Moreover, in the study conducted in Hong Kong, 19% out of 500 respondents showed signs of depression in the spring of 2020 [37]. Interestingly, another study showed that the occurrence rate of depression was strongly associated with the time spent on COVID-19 related reports. The depression rate was 17.8% among people who spent less than five minutes daily on COVID-19 related news and oscillated near 28% among individuals who followed the topics daily for one hour [38]. As presented in our study, the high interest in inquiries about “depression” shortly after the pandemic outbreak might mimic this public interest. The WHO advises to minimize listening to news related to the COVID-19 pandemic that may cause anxiety. Only trusted sources should be followed to gain more information on the protection against the virus [39].

Insomnia is a serious health problem associated with a high psychological burden and is also considered as one of the symptoms of depression. A Greek scientific report revealed that 37.6% of 2,363 study participants had sleep problems. Women and urban dwellers were more likely to have sleep problems, while younger persons did not show this significant trend. Elevated insomnia scores occurred in subjects who were unsure whether they or their family members were infected with the virus. Finally, according to the analysis, higher levels of future uncertainty, COVID-19-related distress, loneliness, and depressive symptoms were predictors of insomnia [40].

Moreover, during the COVID-19 pandemic, sleep problems affected approximately 40% of the general population and health care providers. The prevalence of sleep problems was higher in patients suffering from COVID-19 disease [41]. These outcomes agree with another study, where insomnia was referred to as “coronasomnia/COVID-somia”. Together with sleep fragmentation and nightmares, it became more frequent in the general population during the COVID-19 pandemic [42]. Interestingly, in a French study conducted by Kokou–Kpolou et al., in which diagnostic criteria for insomnia were met by 19.1% of the respondents, results were similar to the outcomes from the Italian population [43]. However, these percentage ratios were lower than in the study conducted by Voitsidis et al. [40], where sleep problems affected 37.6% of the participants. French authors stated that loneliness and sadness related to COVID-19 were significant predictors of clinical insomnia, in addition to pre-existing mental illness. According to them, sleep problems should be considered one of the emerging mental health problems of the COVID-19 pandemic [44].

In another study, the increase in search queries for insomnia from 20 March to 19 April 2020 was positively correlated to the number of COVID-19 deaths. However, the contrary is the case in searches on depression where there was no correlation with the COVID-19 deaths [45]. The GT forecast predicts an increase in insomnia queries by 5.2%. Our results align with recent studies where the longitudinal sleep problems, even after the pandemic, were also predicted [46].

Loneliness is described as the perceived disparity between the actual and expected social relationships [47]. According to conducted studies, the COVID-19 pandemic will significantly impact people’s well-being and health. The findings of Brodeur et al. indicate that mental health has been severely affected by the COVID-19 pandemic. The authors observed a significant increase in online searches for loneliness [48]. Children and adolescents are more likely to experience depression after isolation; the longer the loneliness lasted, the more severe were the mental health problems [49]. Loneliness and forced social isolation affect older adults as well and contribute to their physical and mental health [50]. Shah et al. suggest that in order to reduce loneliness during lockdowns, people should access digital technology to maintain contact with others [51]. The broader use of digital technology to connect with loved ones may partially explain the decrease in the near future interest in the phrase “loneliness”, predicted in our study. Moreover, it might be explained as natural habituation to difficult conditions [52] accompanied by the gradual removal of lockdown and social distancing rules that had been announced due to the anticontagion policies.

Our study predicts more significant interest in “psychiatry” and near stable interest in “psychologist” expressions in the near future. The challenging times of the COVID-19 pandemic have shown a growing public health burden due to more psychiatric and mental disorders [53,54], which might explain the predicted increase in “psychiatrist” expression. However, the negative impact of the pandemic on mental health can push psychiatric services to the limit [55]. Nevertheless, adequate support should be provided to help those struggling with mental health problems during the pandemic. As Lancet Psychiatry reports [56], conducting research during a public health crisis is challenging, so an ambitious mental health program is needed. A large sample of information obtained from Google search engine users worldwide could assist in anonymous, timely data collection without generating high costs. Such a survey may be carried out to identify and assess social needs in multiple fields, as well as predict the mental health outlook for a given population. During the COVID-19 pandemic, the ability to track emerging trends in mental health symptoms can guide public health responses. As changes in mental health can escalate, ongoing surveillance is important. GT can be used to predict population mental health needs [14].

This analysis has some limitations—the study used only one search engine and a global approach survey. Google search engine is the largest [57], and the phrases asked in the English language refer to numerous countries worldwide. We did not translate search queries because a comprehensive understanding of each language is required to preserve the exact meaning of each search term. Since our study was not specifically designed to compare search interest levels between different countries, this problem of a multilanguage analysis does not affect our conclusion. However, there might be differences between countries with different measures of COVID-19 restrictions. An unavoidable aspect of such studies may also be overlapping phrases [58] or errors in the approach to questions asked by a single Google search user. On the other hand, the 5.4 billion Google searches per day and the online search for information colloquially known as “google it” show the popularity and the extensive global use of this search engine [57]. The ability to quickly track recent population behaviour might become an advantage, and in such an environment, GT is a powerful tool for tracing public interest. Other studies have used GT to monitor public concerns related to medical, social, and political issues [12,59,60]. This type of analysis is a modern alternative to smaller cross-sectional surveys with fewer data and longer time lag. Finally, the exact design of Google’s algorithms is not revealed by the company. In the conducted study, phrases were attentively selected to reflect the purpose of the study and the topic we were to investigate.

## 5. Conclusions

In the short term, the level of queries asked in GT regarding “depression” and “loneliness” are expected to decrease. This can be explained by the active vaccination of the population and the gradual lockdown abolition. Moreover, at the same time, interest in “insomnia” and “psychiatrist” queries is expected to increase while the interest in “psychologists” expression tends to have stable values. The results can be used to develop therapeutic strategies and social policies to support people with mental health problems during the COVID-19 pandemic.

## Figures and Tables

**Table 1:** The depression’s hybrid model accuracy measures.

	ME	RMSE	MAE	MPE	MAPE	ACF1	Theil’s U
M1	−0.17	3.56	2.49	−0.56	3.42	0.10	0.61
M2	−0.17	3.41	2.31	−0.51	3.16	0.05	0.58
M3	−0.18	3.48	2.31	−0.52	3.17	0.03	0.60

A hybrid model with equal weights (M1); hybrid model weighting by in-sample error (M2); hybrid model weighting by in-sample error with parameter tuning (M3); margin of error (ME); root mean square deviation (RMSE); mean absolute error (MAE); mean percentage error (MPE); mean absolute percentage error (MAPE); autocorrelation of errors at lag 1 (ACF1); uncertainty coefficient (Theil’s U).

**Table 2:** The M2 model’s component weights for “depression” score forecasting.

	Auto Arima	Thetam	Nnetar	Stlm	Tbats
M2	0.24	0.11	0.26	0.25	0.14

**Table 3:** Insomnia’s hybrid model accuracy measures.

	ME	RMSE	MAE	MPE	MAPE	ACF1	Theil’s U
M1	0.13	3.32	2.61	−0.03	3.37	0.02	0.57
M2	0.06	1.94	1.51	−0.05	1.96	0.01	0.33
M3	0.06	1.80	1.39	−0.04	1.80	0.02	0.31

A hybrid model with equal weights (M1); hybrid model weighting by in-sample error (M2); hybrid model weighting by in-sample error with parameter tuning (M3); margin of error (ME); root mean square deviation (RMSE); mean absolute error (MAE); mean percentage error (MPE); mean absolute percentage error (MAPE); autocorrelation of errors at lag 1 (ACF1); uncertainty coefficient (Theil’s U).

**Table 4:** The M3 model’s component weights for insomnia score forecasting.

	Auto Arima	Thetam	Nnetar	Stlm	Tbats
M3	0.10	0.09	0.64	0.11	0.06

**Table 5:** Loneliness’s hybrid model accuracy measures.

	ME	RMSE	MAE	MPE	MAPE	ACF1	Theil’s U
M1	0.01	4.07	3.1	−0.49	5.18	0.08	0.55
M2	<0.01	2.04	1.57	−0.27	2.66	0.02	0.28
M3	<0.01	1.93	1.48	−0.25	2.49	0.03	0.26

A hybrid model with equal weights (M1); hybrid model weighting by in-sample error (M2); hybrid model weighting by in-sample error with parameter tuning (M3); margin of error (ME); root mean square deviation (RMSE); mean absolute error (MAE); mean percentage error (MPE); mean absolute percentage error (MAPE); autocorrelation of errors at lag 1 (ACF1); uncertainty coefficient (Theil’s U).

**Table 6:** The M3 model’s component weights for loneliness score forecasting.

	Auto Arima	Thetam	Nnetar	Stlm	Tbats
M3	0.09	0.08	0.69	0.09	0.05

**Table 7:** Psychologist’s hybrid model accuracy measures.

	ME	RMSE	MAE	MPE	MAPE	ACF1	Theil’s U
M1	0.05	4.27	3.23	−0.47	4.52	0.15	0.53
M2	0.03	3.83	2.89	−0.39	4.01	0.08	0.47
M3	−0.04	3.89	2.87	−0.50	4.00	0.08	0.48

A hybrid model with equal weights (M1); hybrid model weighting by in-sample error (M2); hybrid model weighting by in-sample error with parameter tuning (M3); margin of error (ME); root mean square deviation (RMSE); mean absolute error (MAE); mean percentage error (MPE); mean absolute percentage error (MAPE); autocorrelation of errors at lag 1 (ACF1); uncertainty coefficient (Theil’s U).

**Table 8:** Model’s M3 component weights for “psychologist” score forecasting.

	Auto Arima	Thetam	Nnetar	Stlm	Tbats
M3	0.22	0.11	0.32	0.21	0.14

**Table 9:** The psychiatrist’s hybrid model accuracy measures.

	ME	RMSE	MAE	MPE	MAPE	ACF1	Theil’s U
M1	0.10	5.39	4.18	−0.31	5.17	0.01	0.68
M2	0.10	5.31	4.11	−0.31	5.08	0.01	0.67
M3	0.18	5.37	4.08	−0.22	5.05	−0.01	0.68

A hybrid model with equal weights (M1); hybrid model weighting by in-sample error (M2); hybrid model weighting by in-sample error with parameter tuning (M3); margin of error (ME); root mean square deviation (RMSE); mean absolute error (MAE); mean percentage error (MPE); mean absolute percentage error (MAPE); autocorrelation of errors at lag 1 (ACF1); uncertainty coefficient (Theil’s U).

**Table 10:** The M3 model’s component weights for “psychiatrist” score forecasting.

	Auto Arima	Thetam	Nnetar	Stlm	Tbats
M3	0.21	0.19	0.22	0.25	0.13

## Data Availability

The data presented in this study are available on request from the corresponding author.
